# 5,6-Dihydro-2*H*-1,3-dithiolo[4,5-*b*][1,4]dioxine-2-thione

**DOI:** 10.1107/S1600536811017417

**Published:** 2011-05-14

**Authors:** Guan-nan Wang, Xun-wen Xiao, Tongjiang Cai, Qin Huang

**Affiliations:** a Department of Chemistry and Chemical Engineering, Taiyuan University of Technology, Taiyuan, People’s Republic of China; b Department of Chemical Engineering, Ningbo University of Technology, Cuibai Road 89, Ningbo, People’s Republic of China

## Abstract

The title mol­ecule, C_5_H_4_O_2_S_3_, consists of a planar [mean deviation = 0.020 (1) Å] 1,3-dithiole-2-thione unit with an ethyl­enedi­oxy group in the 4,5-positions. The dioxine ring is in a twist-chair conformation.

## Related literature

For related structures, see: Kanchanadevi *et al.* (2010 ▶); Rizvi *et al.* (2010 ▶); Suzuki *et al.* (1989 ▶); Xu *et al.* (2009 ▶); Sugumar *et al.* (2008 ▶). For the synthesis of the title compound, see: Hartke & Lindenblatt (1990 ▶); Suzuki *et al.* (1989 ▶).
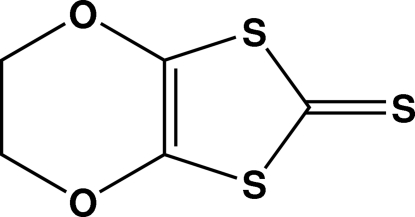


## Experimental

### 

#### Crystal data

C_5_H_4_O_2_S_3_
*M* *_r_* = 192.26
Monoclinic, 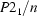

*a* = 5.4645 (8) Å
*b* = 13.430 (2) Å
*c* = 9.8189 (16) Å
β = 91.294 (3)°
*V* = 720.41 (19) Å^3^
*Z* = 4
Mo *K*α radiation
μ = 0.96 mm^−1^
*T* = 223 K
0.55 × 0.20 × 0.20 mm

#### Data collection

Rigaku Saturn diffractometer
Absorption correction: multi-scan (*REQAB*; Jacobson, 1998 ▶) *T*_min_ = 0.605, *T*_max_ = 0.826
3605 measured reflections
1332 independent reflections
1199 reflections with *I* > 2σ(*I*)
*R*_int_ = 0.021

#### Refinement

*R*[*F* ^2^ > 2σ(*F* ^2^)] = 0.031
*wR*(*F* ^2^) = 0.083
*S* = 1.17
1332 reflections
92 parameters
H-atom parameters constrained
Δρ_max_ = 0.39 e Å^−3^
Δρ_min_ = −0.41 e Å^−3^

### 

Data collection: *CrystalClear* (Rigaku, 2005 ▶); cell refinement: *CrystalClear*; data reduction: *CrystalStructure* (Rigaku, 2005 ▶); program(s) used to solve structure: *SHELXS97* (Sheldrick, 2008 ▶); program(s) used to refine structure: *SHELXL97* (Sheldrick, 2008 ▶); molecular graphics: *ORTEP-3* (Farrugia, 1997 ▶); software used to prepare material for publication: *SHELXTL* (Sheldrick, 2008 ▶).

## Supplementary Material

Crystal structure: contains datablocks I, global. DOI: 10.1107/S1600536811017417/aa2007sup1.cif

Structure factors: contains datablocks I. DOI: 10.1107/S1600536811017417/aa2007Isup2.hkl

Supplementary material file. DOI: 10.1107/S1600536811017417/aa2007Isup3.cml

Additional supplementary materials:  crystallographic information; 3D view; checkCIF report

## Figures and Tables

**Fig. 1.**
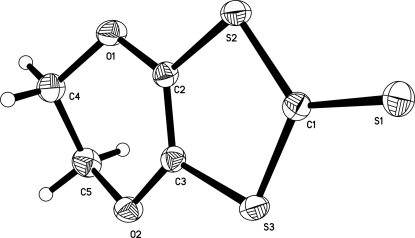
The molecular structure of the title compound. Displacement ellipsoids are drawn at the 30% probability level.

**Fig. 2.**
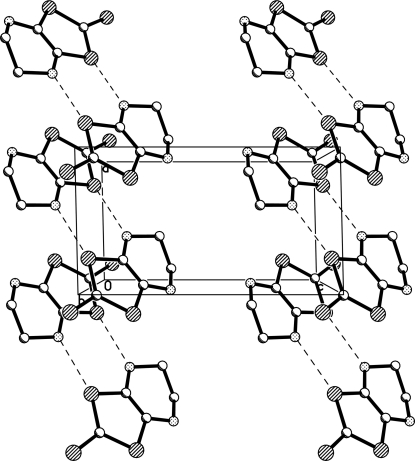
The crystal packing diagram view along the crystallographic b axis.

## supplementary crystallographic information

### Comment

The title compound is a precursor to ethylenedioxotetrathiafulvalene(EDO-TTF). EDO-TTF has attracted much interest due to ultrafast gigantic photo-response in organic salts (EDO-TTF)_2_PF_6_.

In the title compound (Fig.1), the five-membered ring and attached S and O atoms are essentially coplanar, mean deviation from the mean plane 0.020 (1) Å. The C—S bond lengths range from 1.728 to 1.748 Å, it is smaller than that typical of C—S bond lengths, 1.82 Å, suggesting a degree of conjugation in the dithiole-2-thione system. The dioxine ring is in a twist-chair conformation.

### Experimental

The C_5_H_4_S_3_O_2_ was prepared according to the literature (Suzuki *et al.*, 1989).Yellow crystals were obtained from slow evaporation of a dichloromethane solution at room temperature.

### Refinement

The H atoms were included at geometrically idealized positions and refined in riding-model approximation with the following constraints: C—H distances were set to 0.98 Å for methylene H-atoms, with *U*_iso_(H) = 1.2(C) *U*_eq_ of the carrier atoms.

### Crystal data

**Table:**

C_5_H_4_O_2_S_3_	*F*(000) = 392
*M**_r_* = 192.26	*D*_x_ = 1.773 Mg m^−^^3^
Monoclinic, *P*2_1_/*n*	Mo *K*α radiation, λ = 0.71075 Å
Hall symbol: -P 2yn	Cell parameters from 3311 reflections
*a* = 5.4645 (8) Å	θ = 3.0–27.5°
*b* = 13.430 (2) Å	µ = 0.96 mm^−^^1^
*c* = 9.8189 (16) Å	*T* = 223 K
β = 91.294 (3)°	Block, yellow
*V* = 720.41 (19) Å^3^	0.55 × 0.20 × 0.20 mm
*Z* = 4	

### Data collection

**Table:**

Rigaku Saturn diffractometer	1332 independent reflections
Radiation source: fine-focus sealed tube	1199 reflections with *I* > 2σ(*I*)
graphite	*R*_int_ = 0.021
Detector resolution: 14.63 pixels mm^-1^	θ_max_ = 25.5°, θ_min_ = 3.0°
ω scans	*h* = −6→6
Absorption correction: multi-scan (REQAB; Jacobson, 1998)	*k* = −14→16
*T*_min_ = 0.605, *T*_max_ = 0.826	*l* = −11→9
3605 measured reflections	

### Refinement

**Table:**

Refinement on *F*^2^	Primary atom site location: structure-invariant direct methods
Least-squares matrix: full	Secondary atom site location: difference Fourier map
*R*[*F*^2^ > 2σ(*F*^2^)] = 0.031	Hydrogen site location: inferred from neighbouring sites
*wR*(*F*^2^) = 0.083	H-atom parameters constrained
*S* = 1.17	*w* = 1/[σ^2^(*F*_o_^2^) + (0.0486*P*)^2^ + 0.013*P*] where *P* = (*F*_o_^2^ + 2*F*_c_^2^)/3
1332 reflections	(Δ/σ)_max_ < 0.001
92 parameters	Δρ_max_ = 0.39 e Å^−^^3^
0 restraints	Δρ_min_ = −0.41 e Å^−^^3^

### Special details

**Table:**

Geometry. All s.u.'s (except the s.u. in the dihedral angle between two l.s. planes) are estimated using the full covariance matrix. The cell s.u.'s are taken into account individually in the estimation of s.u.'s in distances, angles and torsion angles; correlations between s.u.'s in cell parameters are only used when they are defined by crystal symmetry. An approximate (isotropic) treatment of cell s.u.'s is used for estimating s.u.'s involving l.s. planes.
Refinement. Refinement of *F*^2^ against ALL reflections. The weighted *R*-factor *wR* and goodness of fit *S* are based on *F*^2^, conventional *R*-factors *R* are based on *F*, with *F* set to zero for negative *F*^2^. The threshold expression of *F*^2^ > σ(*F*^2^) is used only for calculating *R*-factors(gt) *etc*. and is not relevant to the choice of reflections for refinement. *R*-factors based on *F*^2^ are statistically about twice as large as those based on *F*, and *R*- factors based on ALL data will be even larger.

### Fractional atomic coordinates and isotropic or equivalent isotropic displacement parameters (Å^2^)

**Table:**

	*x*	*y*	*z*	*U*_iso_*/*U*_eq_	
S1	1.15131 (11)	0.20574 (4)	1.05803 (6)	0.0394 (2)	
S2	0.77835 (10)	0.36220 (4)	0.99990 (5)	0.03292 (19)	
S3	1.18427 (10)	0.33798 (4)	0.81841 (6)	0.03387 (19)	
O1	0.6189 (3)	0.51707 (11)	0.85167 (16)	0.0381 (4)	
O2	1.0238 (3)	0.49167 (11)	0.66988 (16)	0.0367 (4)	
C1	1.0418 (4)	0.29782 (14)	0.9629 (2)	0.0282 (5)	
C2	0.7954 (4)	0.44643 (15)	0.8651 (2)	0.0276 (4)	
C3	0.9812 (4)	0.43492 (14)	0.7810 (2)	0.0280 (5)	
C4	0.6183 (4)	0.55689 (18)	0.7147 (2)	0.0412 (6)	
H4A	0.5199	0.6179	0.7115	0.049*	
H4B	0.5419	0.5086	0.6523	0.049*	
C5	0.8699 (5)	0.57960 (16)	0.6687 (2)	0.0413 (6)	
H5A	0.8607	0.6069	0.5761	0.050*	
H5B	0.9437	0.6303	0.7286	0.050*	

### Atomic displacement parameters (Å^2^)

**Table:**

	*U*^11^	*U*^22^	*U*^33^	*U*^12^	*U*^13^	*U*^23^
S1	0.0427 (4)	0.0365 (3)	0.0392 (4)	0.0112 (2)	0.0020 (3)	0.0078 (2)
S2	0.0318 (3)	0.0340 (3)	0.0333 (3)	0.0071 (2)	0.0086 (2)	0.0068 (2)
S3	0.0289 (3)	0.0393 (3)	0.0337 (3)	0.0073 (2)	0.0071 (2)	0.0031 (2)
O1	0.0372 (8)	0.0368 (8)	0.0406 (9)	0.0126 (7)	0.0095 (7)	0.0107 (7)
O2	0.0364 (8)	0.0400 (9)	0.0340 (9)	0.0041 (7)	0.0079 (7)	0.0105 (6)
C1	0.0288 (11)	0.0264 (11)	0.0295 (11)	0.0008 (8)	0.0001 (9)	−0.0033 (8)
C2	0.0276 (10)	0.0260 (10)	0.0293 (11)	0.0013 (8)	0.0010 (8)	0.0018 (8)
C3	0.0256 (10)	0.0289 (10)	0.0296 (11)	−0.0021 (8)	−0.0003 (9)	0.0015 (8)
C4	0.0417 (13)	0.0424 (12)	0.0394 (13)	0.0116 (11)	0.0019 (10)	0.0134 (10)
C5	0.0499 (14)	0.0354 (12)	0.0387 (13)	0.0029 (10)	0.0026 (11)	0.0091 (10)

### Geometric parameters (Å, °)

**Table:**

S1—C1	1.654 (2)	O2—C5	1.450 (3)
S2—C1	1.725 (2)	C2—C3	1.332 (3)
S2—C2	1.746 (2)	C4—C5	1.488 (3)
S3—C1	1.720 (2)	C4—H4A	0.9800
S3—C3	1.744 (2)	C4—H4B	0.9800
O1—C2	1.357 (2)	C5—H5A	0.9800
O1—C4	1.447 (3)	C5—H5B	0.9800
O2—C3	1.356 (2)		
			
C1—S2—C2	96.07 (10)	O1—C4—C5	112.07 (18)
C1—S3—C3	96.32 (9)	O1—C4—H4A	109.2
C2—O1—C4	109.56 (15)	C5—C4—H4A	109.2
C3—O2—C5	110.75 (15)	O1—C4—H4B	109.2
S1—C1—S3	122.39 (12)	C5—C4—H4B	109.2
S1—C1—S2	123.28 (13)	H4A—C4—H4B	107.9
S3—C1—S2	114.33 (12)	O2—C5—C4	111.73 (18)
C3—C2—O1	124.84 (18)	O2—C5—H5A	109.3
C3—C2—S2	116.70 (16)	C4—C5—H5A	109.3
O1—C2—S2	118.45 (14)	O2—C5—H5B	109.3
C2—C3—O2	125.52 (18)	C4—C5—H5B	109.3
C2—C3—S3	116.52 (16)	H5A—C5—H5B	107.9
O2—C3—S3	117.97 (14)		
			
C3—S3—C1—S1	−177.35 (13)	O1—C2—C3—S3	−179.51 (15)
C3—S3—C1—S2	2.19 (13)	S2—C2—C3—S3	−0.7 (2)
C2—S2—C1—S1	177.05 (14)	C5—O2—C3—C2	11.6 (3)
C2—S2—C1—S3	−2.48 (13)	C5—O2—C3—S3	−168.64 (15)
C4—O1—C2—C3	17.2 (3)	C1—S3—C3—C2	−0.90 (19)
C4—O1—C2—S2	−161.53 (15)	C1—S3—C3—O2	179.31 (15)
C1—S2—C2—C3	1.93 (18)	C2—O1—C4—C5	−45.5 (3)
C1—S2—C2—O1	−179.19 (16)	C3—O2—C5—C4	−39.8 (2)
O1—C2—C3—O2	0.3 (3)	O1—C4—C5—O2	59.5 (3)
S2—C2—C3—O2	179.05 (15)		

## References

[bb1] Farrugia, L. J. (1997). *J. Appl. Cryst.* **30**, 565.

[bb2] Hartke, K. & Lindenblatt, T. (1990). *Synthesis*, pp. 281–283.

[bb3] Jacobson, R. (1998). *REQAB* Private communication to the Rigaku Corporation, Tokyo, Japan.

[bb4] Kanchanadevi, J., Dhayalan, V., Mohanakrishnan, A. K., Anbalagan, G., Chakkaravarthi, G. & Manivannan, V. (2010). *Acta Cryst.* E**66**, o3264–o3265. 10.1107/S1600536810047343 PMC3011594 21589548

[bb5] Rigaku (2005). *CrystalClear* and *CrystalStructure* Rigaku Corporation, Tokyo, Japan.

[bb6] Rizvi, S. U. F., Siddiqui, H. L., Hussain, T., Azam, M. & Parvez, M. (2010). *Acta Cryst.* E**66**, o744. 10.1107/S1600536810007464 PMC2983824 21580589

[bb7] Sheldrick, G. M. (2008). *Acta Cryst.* A**64**, 112–122. 10.1107/S0108767307043930 18156677

[bb8] Sugumar, P., Ranjith, S., Clement, J. A., Mohanakrishnan, A. K. & Ponnuswamy, M. N. (2008). *Acta Cryst.* E**64**, o1049. 10.1107/S1600536808012324 PMC2961437 21202568

[bb9] Suzuki, T., Yamochi, H. & Srdanov, G. (1989). *J. Am. Chem. Soc.* **111**, 3108–3109.

[bb10] Xu, J., Xu, H., Quan, J., Sha, F. & Yao, C. (2009). *Acta Cryst.* E**65**, o668. 10.1107/S1600536809007156 PMC2968817 21582412

